# Correction: Transient increased risk of influenza infection following RSV infection in South Africa: findings from the PHIRST study, South Africa, 2016–2018

**DOI:** 10.1186/s12916-024-03355-6

**Published:** 2024-03-21

**Authors:** Naomi R. Waterlow, Jackie Kleynhans, Nicole Wolter, Stefano Tempia, Rosalind M. Eggo, Orienka Hellferscee, Limakatso Lebina, Neil Martinson, Ryan G. Wagner, Jocelyn Moyes, Anne von Gottberg, Cheryl Cohen, Stefan Flasche

**Affiliations:** 1 Centre for Mathematical Modelling of Infectious Disease, School of Hygiene and Tropical Medicine, London, UK; 2 Centre for Respiratory Diseases and Meningitis, National Institute for Communicable Diseases, Johannesburg, South Africa; 3 School of Public Health, Faculty of Health Sciences, University of the Witwatersrand, Johannesburg, South Africa; 4 School of Pathology, Faculty of Medicine, University of the Witwatersand, Johannesburg, South Africa; 5 Perinatal HIV Research Unit, University of the Witwatersrand, Johannesburg, South Africa; 6 Africa Health Research Institute, Durban, South Africa; 7 John Hopkins University Center for TB Research, Baltimore, MD USA; 8 School of Public Health, Faculty of Health Sciences, South African Medical Research Council/Rural Public Health and Health Transitions Research Unit (Agincourt), University of the Witwatersrand, Johannesburg, South Africa

**Correction** **:** **BMC Med 21, 441 (2023)**

**https://doi.org/10.1186/s12916-023-03100-5**

The production team handling this article initially mistakenly omitted corresponding authorship from co-corresponding author, Naomi Waterlow which has since been restored.

